# Posttraumatic growth in people after stroke in the light of selected health-promoting psychological predictors

**DOI:** 10.1192/j.eurpsy.2026.11827

**Published:** 2026-07-31

**Authors:** P. Dębski, M. Garczarczyk, W. Nawara, A. Siostrzonek-Sergiel, H. Czyż, D. Turska-Czyż, M. Adamczyk, W. Madejczyk, J. Smolarczyk, M. Piegza, P. Gorczyca

**Affiliations:** 1 Department of Psychiatry, Medical University of Silesia, Katowice; 2 Faculty of Psychology, Humanitas University, Sosnowiec; 3 WSB Merito University in Poznań, Chorzów; 4 Department of Psychoprophylaxis, Medical University of Silesia, Katowice, Poland

## Abstract

**Introduction:**

The concept of posttraumatic growth refers to positive changes following the experience of a traumatic event, including illness, which can lead to an increased appreciation of life in all its aspects. Numerous correlates of posttraumatic growth have been identified, including phenomena from the field of social psychology, such as perceived social support and psychological resilience. It seems particularly important to examine the predictors of posttraumatic growth in stroke people at risk for disability and the development of mental disorders, including depression.

**Objectives:**

The aim of the study was to assess potential psychological predictors of posttraumatic growth – sense of meaning in life, perceived social support and psychological resilience in people after stroke.

**Methods:**

A total of 141 stroke survivors (mean age 45.48 ± 11.35 SD years) participated in the study, including 100 women and 41 men. The Posttraumatic Growth Inventory (PTGI), the Multidimensional Scale of Perceived Social Support (MSPSS), the Ego Resiliency Scale (ER-89), and the Meaning in Life Questionnaire (MLQ) were used for the statistical analysis. Pearson’s r coefficient and backward stepwise multiple regression were used.

**Results:**

Among stroke survivors, the intensity of posttraumatic growth was significantly and positively correlated with scores on meaning in life, perceived social support, and psychological resilience. The strength of the relationships was mostly moderate or strong. Across all subscales of posttraumatic growth—changes in perception of self (β = 0.396; p= 0.000), changes in relationships with others (β = 0.462; p= 0.000), appreciating life (β = 0.636; p= 0.000), and spiritual changes (β = 0.509; p= 0.000), the presence of meaning in life proved to be the strongest positive predictor.

**Conclusions:**

Sense of meaning in life, perceived social support, and psychological resilience are positive correlates of posttraumatic growth among stroke survivors. The strongest predictor was the sense of meaning in life.

**Disclosure of Interest:**

None Declared

